# Evaluation of the safety, tolerance and efficacy of 1-year consumption of infant formula supplemented with *Lactobacillus fermentum* CECT5716 Lc40 or *Bifidobacterium breve* CECT7263: a randomized controlled trial

**DOI:** 10.1186/s12887-019-1753-7

**Published:** 2019-10-21

**Authors:** J. Maldonado, M. Gil-Campos, J. A. Maldonado-Lobón, M. R. Benavides, K. Flores-Rojas, R. Jaldo, I. Jiménez del Barco, V. Bolívar, A. D. Valero, E. Prados, I. Peñalver, M. Olivares

**Affiliations:** 1 Pediatric Unit, University Hospital Virgen de las Nieves, Granada, Spain; 2 Pediatric Department, University of Granada, Granada, Spain; 3 Biosanitary Research Institute (IBS), Granada, Spain; 4 Maternal and Child Health and Development Network (SAMID), Health Institute Carlos III, Madrid, Spain; 5 Unit of Metabolism and Pediatric Research (IMIBIC), Reina Sofia University Hospital, University of Córdoba, Cordoba, Spain; 6 CIBEROBN, Cordoba, Spain; 7 Biosearch Life, Research Department, Granada, Spain; 8 Pediatric Clinic Roquetas, Roquetas de Mar, Almería, Spain; 9 Andalusian Health Service, Andalusia, Spain; 10 Clinic “Cristo de la Salud” Albolote, Granada, Spain

**Keywords:** Infant formula, Probiotics, Safety, Diarrhoea, Colic

## Abstract

**Background:**

The microorganism present in breast milk, added to other factors, determine the colonization of infants. The objective of the present study is to evaluate the safety, tolerance and effects of the consumption of a milk formula during the first year of life that is supplemented with *L. fermentum* CECT5716 or *Bifidobacterium breve* CECT7263, two strains originally isolated from breast milk.

**Methods:**

A randomized, double blind, controlled, parallel group study including healthy, formula-fed infants was conducted. Two hundred and thirty-six 1-month-old infants were selected and randomly divided into three study groups according to a randomization list. Infants in the control group received a standard powdered infant formula until 12 months of age. Infants in the probiotic groups received the same infant formula but supplemented with *L. fermentum* CECT5716 Lc40 or *B. breve* CECT7263. Main outcome was weigh-gain of infants as safety marker.

**Results:**

One hundred and eighty-nine infants completed the eleven months of intervention (61 in control group, 65 in Lf group and 63 in Bb group). The growth of infants in the three groups was consistent with standards. No significant differences were observed in the main outcome, weight-gain (Control group: 5.77 Kg ± 0.95, Lf group: 5.77 Kg ± 1.31, Bb group: 5.58 Kg ± 1.10; *p* = 0.527). The three milk formulae were well tolerated, and no adverse effects were related to the consumption of any of the formula. Infants receiving *B. breve* CECT7263 had a 1.7 times lower risk of crying than the control group (OR = 0.569, CI 95% 0.568–0.571; *p* = 0.001). On the other hand, the incidence of diarrhoea in infants receiving the formula supplemented with *L. fermentum* CECT5716 was a 44% lower than in infants receiving the control formula (*p* = 0.014). The consumption of this *Lactobacillus* strain also reduced the duration of diarrhoea by 2.5 days versus control group (*p* = 0.044).

**Conclusions:**

The addition of *L. fermentum* CECT5716 Lc40 or *B. breve* CECT7263, two probiotic strains naturally found in breast milk, to infant formulae is safe and induces beneficial effects on the health of infants.

**Trial registration:**

The trial was retrospectively registered in the US Library of Medicine (www.clinicaltrial.gov) with the number NCT03204630. Registered 11 August 2016.

## Background

Breast milk contains physiological microbiota, which contributes to colonization in infants [1, 2]. The microorganisms present in breast milk, as well as the prebiotic factors included in it, determine the colonization of infants and contribute to the differences found between the microbiota of formula-fed and breast-fed infants [3]. Correct, early colonization is a key factor in the process of maturation of infants during the first months [4]. In this sense, studies showing that the first contact with microorganisms triggers a cascade of reactions that are crucial for infant immune system maturation have been particularly relevant [5, 6]. In fact, exposition to microbial dysbiosis early in life has been associated with diseases related to the dysfunction of the immune response, such as allergies [7, 8], type 1 diabetes [9], celiac disease [10] and inflammatory bowel disease [11]. Because of the importance of early colonization on the future health of infants, several strategies to modulate infant colonization are being used in infant nutrition. One of these strategies is the supplementation of infant formula with probiotic microorganisms and prebiotic factors, such as galacto oligosaccharides.

In this context, the use, in infant formula, of bacterial strains originally found in the breast milk of healthy women seems to be a coherent strategy to supply to the formula-fed infants with microorganisms that are naturally provided to breast-fed infants. In line with this strategy, two clinical trials were previously performed in infants with *Lactobacillus fermentum* CECT5716, a probiotic strain originally isolated from breast milk. The studies demonstrated the safety of the probiotic strain, even long term [12, 13]. Moreover, the administration of the strain was associated with a lower incidence of gastrointestinal infections in the infants during the period of intervention, which was corroborated in the two clinical trials performed in the infant population [12, 14].

While it is true that *Lactobacillus* is a common inhabitant in an infant’s intestine, the genus *Bifidobacterium* is one of the most abundant, especially in breast-fed infants [15, 16]. *Bifidobacterium* has been associated with beneficial effects on immune and intestinal function [17, 18]. Since *Bifidobacterium spp* are also found in breast milk, the addition of *Bifidobacterium* strains to infant formula adheres to the strategy to supply formula-fed infants with strains naturally found in breast milk.

To date, a large number of studies have been carried out in this field with very positive results for some of the strains analysed. However, experts still think that more evidence is needed [19, 20]. The objective of the present study is to evaluate the safety, tolerance and effects of the consumption of milk formula supplemented with strains originally isolated from breast milk, *L. fermentum* CECT5716 or *Bifidobacterium breve* CECT7263, during the first year of life.

## Methods

### Study design and medical centers

This is a randomized, double blind, controlled, parallel study with three groups developed in the Paediatrics department of two Spanish hospitals, Hospital Virgen de las Nieves (Granada, Spain) and Hospital Reina Sofía (Córdoba, Spain), two private paediatric clinics (Roquetas de Mar, Almería (Spain) and “Cristo de la Salud”, Granada (Spain)), and 7 public paediatric health centers (Andalusian Health Service, Granada (Spain)).The study adheres to CONSORT guidelines and was retrospectively registered in the US Library of Medicine (www.clinicaltrial.gov) with the number NCT03204630.

### Participants and criteria of inclusion

Healthy infants one-month of age who were exclusively feeding with infant formula were included in the study after the parents or caregivers gave written consent. Subjects were excluded from the study if they had a history of mild or serious gastrointestinal disorders (gastroesophageal reflux, history of chronic diarrhoea or constipation), gastrointestinal surgery, metabolic disorders (lactose intolerance, diabetes), cow’s milk protein allergy, immune deficiency, antibiotic prescription one-week before inclusion and prior use of probiotic-containing-infant formula.

### Sample size calculation

The variable for the calculation of the sample size was the primary outcome, which was the average weight gain of infants between baseline and 120 ± 3 days of age. Taking into account several safety studies in which growth was considered the primary outcome [21, 22] and in accordance with the Scientific Committee for Food Report, the study was designed to have power to detect a difference in weight gain equal to 0.5 standard deviations [23]. Therefore, it was calculated that 63 children would be required in each formula group with a significance level of 5% and a power of 80% (two-sided test). In order to compensate for drop outs during the intervention, the sample size was increased by 25%. Statistical computations in R was performed to calculate sample size needed to test interactions: One formulation was based on the formula proposed per Lu et al. [24] for analysing repeated measures with missing data, and for the 5% level, 80% power and progressively missing 20% of responses and the total sample size per treated group of 76 participants were needed (library longpower from R). The second calculation was performed regarding the sample sized need for obtaining regression coefficients in multiple regression [25]. The implementation of the calculation was performed in R using the library MBESS, and the results obtained was a total of 34 participant per regression coefficient.

### Randomization and blinding

Infants included in the study were randomly allocated into three study groups by using the computer program (SIGESMU®). According to the randomization list each recruiter center received batches of infant formula labelled with the corresponding numbers. Each volunteer received in each visit a batch of infant formula contained enough formula for the next 3 months until the next paediatrician visit. The infant formulae were supplied by Lactalis-Puleva (Granada, Spain) in indistinguishable plain white tins. The blinding of the trial was ensured by a sensorial test of the three formulas by an expert panel that concluded the three products were identical. Paediatricians, parents and researchers only knew the volunteer’s code, not knowing which group belonged. The list of randomization was revealed once the study was completed and the code of each group once the statistical analysis of the data was performed.

### Products of the study and guidelines of consumption

The control formula was a standard powdered infant formula with a nutritional composition in accordance with current EU regulations for both starter and follow-on formula. Probiotic groups received the same formula but supplemented with *L. fermentum* CECT5716 Lc40 in case of Lf group or *B. breve* CECT7263 in case of Bb group. In both cases the concentration dose of probiotic strain was10^7^ cfu/g The concentration of the probiotic in the formula was analysed and confirmed every 6 months. Formulae were consumed by the infants from 1 month of age until the age of 12 months (intervention period). The paediatricians prescribed the amounts of formula per day to be administered to the infants and the guidelines for complementary feeding according to current ESPGHAN guidelines [26].

### Study outcomes and data collection

Average weight gain between baseline (1 month of age) and 4 months of age was the primary outcome of the study. Secondary outcomes included average weight, length and head circumference, incidence of intestinal infections, feeding-related behaviour, adverse effects associated with formula consumption and faecal microbiota. The follow-up visits to the paediatrician were performed at baseline and at the ages of 2, 4, 6, 9 and 12 months.

The paediatrician made the diagnosis of infectious diseases according to specific symptoms and standardized definitions. Gastrointestinal infection was characterized by occurrence of loose or watery stool ≥3 times/day with or without a fever or vomiting [27], and respiratory tract infections were determined in case of presence of abundant mucosity and/or cough during two or more days in a row with or without a fever or the presence of wheezing and/or crepitant with or without fever. Parents received a diary to collect data about incidences in the health of the infants and unscheduled visits with a doctor. Moreover, they received a notebook with questionnaires to be completed every two weeks, in which information regarding the daily number of depositions, behaviour and gastrointestinal discomfort were recorded (Table 1).

**Table 1:** Feeding related parameters

During the last 2 days the number of fecal depositions/day	1 = < 1 time, 2 = 1–3 times, 3 = 4–6 times, 4 = 7–10 times, 5= > 10 times
During the las 2 days the feces color was	1 = yellow 2 = mustard 3 = brown 4 = grey 5 = green
During the last 2 days the consistency of feces was	1 = hard lumps, 2 = sausage with cracks, 3 = soft sausage, 4 = mushy (like porridge),5 = watery
During the last 2 days the infant suffered flatulency	1 = 0 h 2 = < 3 h/day 3 = 3-6 h/day 4 = 6-12 h/day 5= > 12 h/day
During the last 2 days the infant suffered regurgitation	1 = not at all, 2 = regurgitation of small amounts during or shortly after feeding, 3 = larger regurgitation during or shortly after feeding, 4 = minor vomiting with time-lag to prior feeding, 5 = severe vomiting with considerable loss of fluid
During the past 2 days the total sum of sleeping hours in 24 h was on average	1 = < 11 h/day, 2 = 11-14 h/day, 3 = 14–17 h/day, 4 = 17–20 h/day, 5= > 20 h/day
Gender Temper: The infant’s behavior when awake during the last 2 days is best described as	1 = tired, passive, 2 = quiet, watching, 3 = well-balanced, active, 4 = bubbly, fidgety, exited, 5 = disturbed, agitate
Colic symptoms: during the last 2 days the infant has suffered continuous and disconsolate crying episodes	⁯ 1 = not at all, 2 = < 3 h/day, 3 = 3–6 h/day, 4 = 6–12 h/day, 5= > 12 h/day

Four faecal samples were collected simultaneously from every infant at baseline and 4, 6, 9 and 12 months of age, maintained at − 20 °C and processed within 4-weeks. Of the four samples, three were used for parameters determination, and the last one was stored at − 80 °C.

### Faecal bacteria quantification

*Lactobacillus spp., Bifidobacterium spp., Clostridium spp., Bacteroides spp*. and *Escherichia coli* counts were measured by quantitative polymerase chain reaction.

The ATP™ GENOMIC DNA MINI KIT (TISSUE) AGT300 (ATP Biotech Inc., Taipei City 10,683, Taiwan) was used for bacterial DNA isolation. In brief, the colonic content was homogenized in Peptone Water at a concentration of 100 mg/ml under sterile conditions, and 200 μl of the previous suspension was added to an Eppendorf tube (20 mg/ml) for DNA extraction following the instructions of the ATP™ GENOMIC DNA MINI KIT (TISSUE) AGT300 protocol.

DNA quantification was performed by qPCR using SYBR® green as a fluorophore (Quantifast SYBR Green OCR Kit Qiagen Cat. No 204057) and specific primers for each group as previously described by Maldonado-Lobón et al. [13].

### Statistical analysis

Descriptive analysis and bivariate statistical tests per treatment group were performed for baseline characteristics as well as the outcomes. For this analysis, when data were continuous and normality could be assumed, ANOVA was performed using F when data were homogeneous and Welch when equal variances could not be assumed. Categorical variable percentages were calculated, and differences between groups were analysed using the chi-square test or the chi-square exact test in the case of large contingency tables without enough cases by cells of categories. For the outcomes related to an event, the Incidence Rate Ratio (IRR) and Odd Ratio (OR) were calculated.

Statistical models were applied in order to analyse the differences in the responses between treatment groups adjusted by other covariates and factors that may be associated to the change of the responses. The statistical models applied to the primary and secondary outcomes were adjusted by time, age at entry, group of treatment, sex, C-section, having siblings, rotavirus vaccination, breastfeeding prior to intervention and gestational age. Linear Mixed Models were applied for continuous data when the residuals were normally distributed. Poisson Mixed Models were applied when the data recorded were related to the number of events observed, and Logistic Mixed Models were applied when the outcomes to be analysed were binary responses.

Ordinal logistic regression mixed models were applied to the responses of secondary outcomes over time and adjusted by relevant covariates. Ordinal outcomes were, for example, stool frequency, consistency, colic and flatulence, regurgitation symptoms and sleeping hours. Additionally, a multinomial logistic regression mixed model was applied for nominal data, such as faeces colour.

The statistical software used to perform the analysis was R version 3.1. Statistical tests at the 5% significance level (two-tailed) were considered for hypothesis testing.

## Results

### Population

**Fig. 1:**
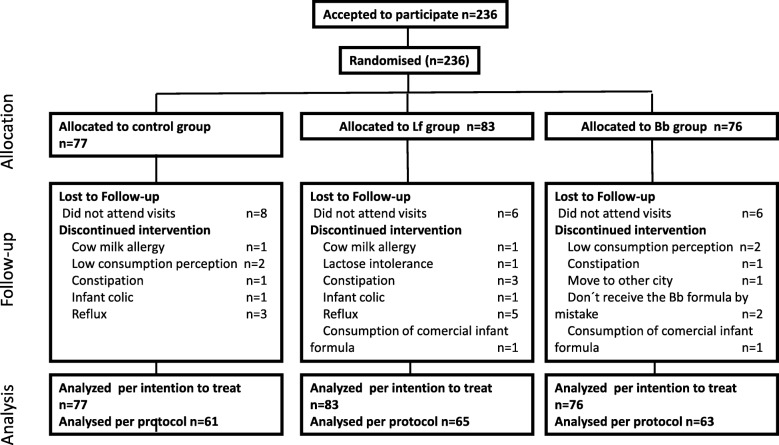
Flow chart of participants

Two hundred and thirty-six infants were included in the study and randomized between April 2011 and July 2012. The intervention ended on June 2013. A flow chart of participants is shown in Fig. 1. Nineteen percent of the infants did not complete the intervention and withdrew from the study. Causes of withdrawal were the perception by the mother that the child did not drink enough milk, changes to another kind of formula because of a cow milk allergy, lactose intolerance, infant colic, reflux or constipation. Two additional infants were excluded because they received a commercial formula and another two infants mistakenly received the formula corresponding to another group of the study. No differences among the groups were detected between the number and causes of withdrawal. The data of all the infants included in the study were analysed (analysis per intention to treat, ITT). For the analysis of Incidence Rate Ratio, in which events accumulated during the 11 months of intervention, only data of infants who completed the intervention were taken into account (per protocol). If statistically significant differences were observed, a second analysis was performed including data of all infants in the study (ITT analysis). In this case, 189 infants who completed the intervention were included in the analysis.

The baseline characteristics of the 236 infants included in the study were analysed. In addition to those related to the infants, they were included mother’s characteristics, variables related to gestation and delivery, and the environment around the infant. All these baseline characteristics were comparable among the study groups (Table 2).

**Table 2:** Baseline characteristics of the subjects that participated in the study

	CG (*n* = 77)	Lf (*n* = 83)	Bb (*n* = 76)
Male/female, n (%)	41/36 (53.2/46.8)	48/35 (57.8/42.2)	39/37 (51.3/48.7)
Age at enrolment (weeks), mean ± SD	3.73 ± 0.93	3.80 ± 1.11	3.75 ± 1.19
Birth weight (kg), mean ± SD	3.21 ± 0.5	3.18 ± 0.4	3.17 ± 0.59
Delivery by cesarean (%)	27.3	26.5	32.9
Gestational age (weeks) mean ± SD	39.0 ± 1.5	39.2 ± 1.3	38.9.2 ± 1.7
Age of mother at birth (years) mean ± SD	34.41 ± 2.3	34.06 ± 1.3	33.93 ± 1.7
Breast feeding (days) mean ± SD	6.13 ± 9.6	4.06 ± 7.8	5.25 ± 8.9
Smoking during pregnancy (%)	16.7	14.5	19.2
Smoking during lactacion (%)	15.6	12.2	17.9
Smoking in the household (%)	42.9	38.6	48.7
Older siblings (%)	61.5	50.6	51.3
Weight of mother (kg) mean ± SD	70.8 ± 13.95	69.8 ± 14.2	70.7 ± 14.1
Pets at home (%)	26.0	32.5	42.1

### Growth of infants

**Fig. 2:**
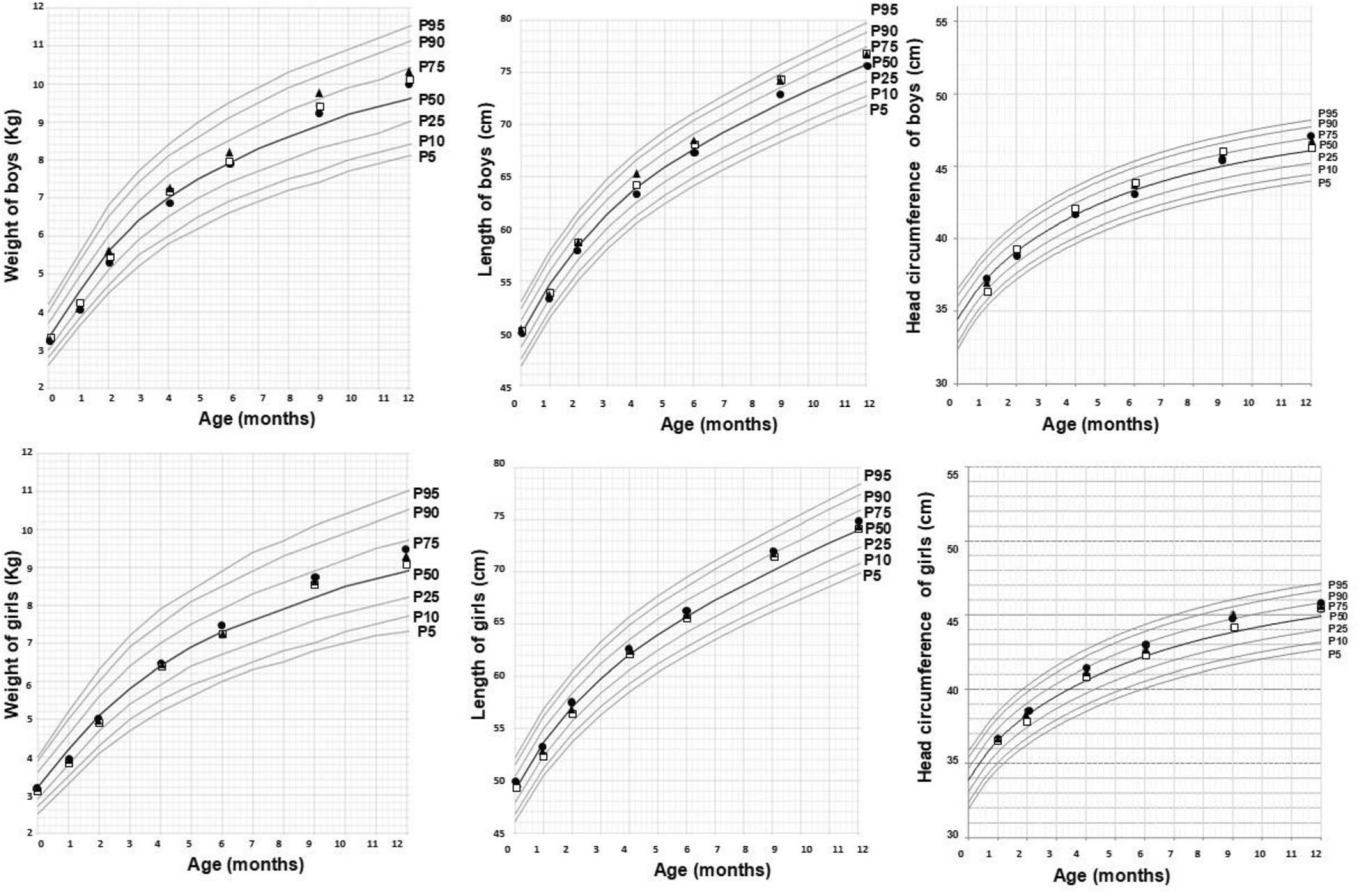
Based on the mean, by gender and group, of the weights, lengths and head circumferences of infants, the corresponding percentile for the mean by boys and girls for each group over time are represented with respect to the standard curves. Black dots show the growth curve, on average, for the Control group. White square curves for the Lf group and triangles for the Bb group. Each of the curves (black lines) is the standard percentile at each point of age of the children

Based on the mean weight, length and head circumference for boys and girls, the corresponding mean for each group over time was represented with respect to the standard percentile curves (Fig. 2). Growth curves for weight, length and head circumference were very similar in the three groups. With respect to weight, values for the three groups remained quite close to one another, around the 50% percentile, until 6 months. After 6 months, the means were between the 50th and 75th percentiles. Similar results were observed for the growth curves of length and head circumference.

Mean weight, length and head circumference for each group were obtained at 1, 2, 4, 6, 9 and 12 months (Table 3). Regarding weight, no significant differences were observed among the groups at any time. To analyse the overall effect, the linear mixed model was adjusted by basal characteristics. The model did not show significant differences between groups (*p* = 0.427 (Lf vs Control); *p* = 0.296 (Bb vs Control)). Moreover, no significant differences were detected in the primary outcome, mean weight gain from 1 to 4 months of age (Control group: 2.67 ± 0.70 Kg; CI 95% 2.50–2.82, Lf group: 2.79 ± 0.79 Kg; CI 95% 2.60–2.98, Bb group: 2.76 ± 0.71 Kg; CI 95% 2.58–2.93; *p* = 0.560). Mean weight gain from 1 to 12 months was also analysed and no significant differences among the groups were observed (Control group: 5.77 ± 0.95 Kg CI 95% 5.53–6.01, Lf group: 5.77 ± 1.31 Kg; CI 95% 5.44–6.10, Bb group: 5.58 ± 1.10 Kg; CI 95% 5.31–5.85; *p* = 0.527).

**Table 3:** Anthropometric measurements at baseline (1 month), 2, 4, 6, 9 and 12 months of age. Values are means ± Standard Deviation (SD)

		1 month			2 months			4 months			6 months			9 months			12 months		
WEIGHT (Kg)		Group Control	Group Lf	Group Bb	Group Control	Group Lf	Group Bb	Group Control	Group Lf	Group Bb	Group Control	Group Lf	Group Bb	Group Control	Group Lf	Group Bb	Group Control	Group Lf	Group Bb
Mean		3.994	4.065	4.038	5.202	5.324	5.170	6.677	6.850	6.754	7.691	7.738	7.554	9.017	9.260	9.001	9.760	9.839	9.625
SD		0.641	0.642	0.697	0.647	0.681	0.664	0.765	0.854	0.835	0.922	1.027	0.911	1.071	1.323	1.157	1.160	1.331	1.226
95% CI	LL	3.850	3.919	3.876	5.055	5.162	5.010	6.497	6.644	6.547	7.468	7.487	7.328	8.752	8.930	8.712	9.467	9.504	9.316
	UL	4.139	4.210	4.201	5.349	5.485	5.329	6.856	7.057	6.961	7.914	7.988	7.779	9.283	9.591	9.290	10.052	10.174	9.933
*p*-value		0.800			0.349			0.455			0.517			0.386			0.619		
LENGTH (cm)		Group Control	Group Lf	Group Bb	Group Control	Group Lf	Group Bb	Group Control	Group Lf	Group Bb	Group Control	Group Lf	Group Bb	Group Control	Group Lf	Group Bb	Group Control	Group Lf	Group Bb
Mean		53.36	53.42	53.23	57.67	57.82	57.59	63.03	64.17	63.18	66.941	67.149	66.700	72.50	73.01	72.98	75.31	75.56	75.47
SD		2.64	2.62	2.55	2.80	3.05	2.59	2.69	3.72	2.22	2.706	3.769	2.564	3.628	3.680	3.67	3.341	3.68	3.27
95% CI	LL	52.76	52.83	52.64	57.04	57.10	56.97	62.40	63.27	62.63	66.286	66.230	66.065	71.601	72.081	72.05	74.48	74.64	74.65
	UL	53.95	54.02	53.83	58.31	58.55	58.21	63.66	65.07	63.73	67.596	68.069	67.335	73.399	73.935	73.90	76.15	76.49	76.30
*p*-value		0.903			0.880			0.049			0.702				0.679			0.919	
HEAD CIRCUMFERENCE (cm)		Group Control	Group Lf	Group Bb	Group Control	Group Lf	Group Bb	Group Control	Group Lf	Group Bb	Group Control	Group Lf	Group Bb	Group Control	Group Lf	Group Bb	Group Control	Group Lf	Group Bb
Mean		36.73	36.68	36.52	38.83	38.80	38.53	41.38	41.46	41.28	43.35	42.85	42.99	45.19	45.23	44.91	46.47	46.10	46.05
SD		1.55	1.29	1.77	1.52	1.25	1.89	1.39	2.20	1.35	1.40	1.37	1.42	1.68	1.81	1.73	1.42	1.44	1.55
95% CI	LL	36.38	36.39	36.11	38.47	38.50	38.07	41.05	40.92	40.94	43.01	42.51	42.63	44.77	44.77	44.48	46.11	45.73	45.64
	UL	37.08	36.98	36.93	39.18	39.10	38.99	41.70	42.00	41.61	43.69	43.18	43.34	45.60	45.69	45.34	46.83	46.46	46.47
*p*-value		0.734			0.473			0.822			0.098			0.527				0.230	

The length of the children was similar throughout the study among the 3 groups. However, at 4 months, infants in the Lf group showed having slightly longer length measurements than those of the control group (*p* = 0.049). This difference was primarily observed in boys (*p* = 0.021). The estimated parameters from the linear mixed model showed no differences for length among experimental groups (*p* = 0.588 (Lf vs Control), *p* = 0.475 (Bb vs Control)). Interestingly, a difference in behaviour or length responses of infants born by C-section was observed depending on the group. In the Control group, infants born by C-section had a lower length than infants born by natural delivery (*p* = 0.042). However, in the Lf and Bb groups, infants born by C-section had higher length responses than infants born by natural delivery (*p* = 0.006 for Lf group and *p* = 0.018 for Bb group).

Finally, no differences were observed in head circumference values among groups (*p* = 0.384 (Lf vs Control; *p* = 0.183 (Bb vs Control)).

### Formula intake, tolerance and adverse effects

No significant differences were found among the study groups in regard to daily intake of formula (Table 4). The daily consumption of the formula corresponded to an average dosage of probiotic bacteria of 1 × 10^9^ cfu/day up to 6 months and 7-8 × 10^8^ cfu/day between 6 and 12 months.

**Table 4:** Formula intake corresponding to amount of milk (mL) reported by parents to be consumed by the infants for each time is showed

INTAKE FORMULA-FED (mL)		2 months	4 months	6 months	9 months	12 months
Control group	Mean	783.659	883.784	730.290	618.750	552.580
	SD	178.112	215.256	273.280	289.056	271.821
	95% CI	727.32–837.54	814.87–951.34	638.83–825.88	535.01–721.85	463.25–653.23
Group Lf	Mean	797.241	890.000	712.140	566.150	486.800
	SD	199.372	165.901	202.052	131.699	181.216
	95% CI	723.46–868.61	826.7–949.25	640.01–788.85	513.86–616.53	425.21–556.8
Group Bb	Mean	851.250	895.517	707.590	567.590	532.220
	SD	211.946	225.730	223.442	135.330	170.166
	95% CI	784.75–931.55	813.82–984.11	633.1–794.13	516.13–622.4	472.59–598.15
	*p*-value	0.627	0.603	0.988	0.998	0.351

No adverse effects associated to supplementation with *L. fermentum* CECT5716 and *B. breve* CECT7263 were detected during the study.

Some withdrawals were related to symptoms which might have been related to the tolerance of the formula, such as reflux, infant colic, constipation or low consumption of the formula (Fig. 1). However, no significant differences in the dropout rates among groups were detected and incidences were in line or even below that those in general population; therefore, symptoms could not be related to the supplementation with *L. fermentum* CECT5716 or *B. breve* CECT7263 and paediatricians considered three formula of the study well tolerated.

Parameters related to tolerance and intestinal function were also evaluated based on the questionnaire explained in methods section (Table 1).

**Fig. 3:**
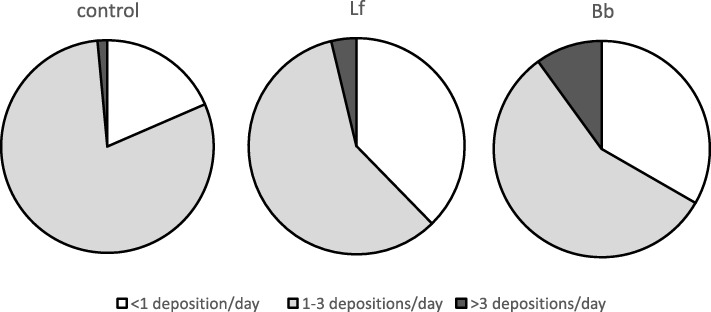
Proportions in each group of infants showing < 1 faecal deposition/day (white), 1–3 faecal depositions/day (grey) or > 3 faecal depositions/day

Faecal depositions: most of the infants showed a stool frequency of 1–3 depositions per day. In general, the probability of an infant to have 1–3 faecal depositions/day is 0.945; in contrast, the probability of having a frequency of less than once a day is 0.095. Differences among the groups were observed during the first month of intervention (*p* = 0.015). For this period, in the Control group, 80% of infants showed a frequency of depositions between 1 and 3 times per day, in contrast with 57–59% of infants in the probiotic groups (Fig. 3). An interaction with the type of delivery was observed. Infants born by natural delivery had a higher risk of having lower frequency of faecal depositions when they were in the Lf or Bv group in comparison with infants from the Control group, whose risk was lower (OR = 0.411 for Lf group *p* = 0.011; OR = 0.404 for group Bv, *p* = 0.009). However, for infants born by C-section, the opposite occurred. For infants born by C-section, the odds of having stools more often was 3.07 and 1.66 times higher in the Lf and Bb groups than in the Control group (*p* = 0.002 Lf vs. Control group; *p* = 0.022 Bb vs. control group).

Faeces colour: yellow, green or grey colours were more likely to be observed at the beginning of the trial. As the trial progressed, the faeces colour was more likely brown. Brown colour was more likely to occur in infants from the Lf and Bb groups than in infants in the Control group (OR = 1.595 for Lf group, p = 0.002 vs. Control group; OR = 1.639 for Bb group, *p* = 0.001 vs. Control group).

Stool consistency: the percentage of infants showing higher stool consistency increased with time (*p* < 0.001). No differences were observed between the Control group and Bb group (*p* = 0.270). However, the behaviour of infants in the Lf group changed with time (p = 0.001), and while it was observed that the risk of having softer stools was 1.89 times lower in the Lf group than in the control group at the beginning of the study, after four months, this risk changed, and the odds of softer consistency was 1.6 times higher in the Lf group than in the Control group (*p* = 0.015).

Symptoms of gastrointestinal discomfort associated with the diet were evaluated. Infants born by C-section had a higher risk of suffering from more flatulence in comparison with infants born by natural delivery (OR = 2.022; *p* = 0.038). As the study progressed, the risk of suffering from flatulence decreased in all of the groups; however, for the Lf group, the decrease throughout the study was more pronounced (OR = 0.658; p = 0.038). At the beginning of the study and during the first two months, more than 70% of infants suffered from regurgitation, which was mild in most cases. As the study progressed over time, the frequency of regurgitation decreased to around 5% at the end of intervention (*p* = 0.000). No differences were observed among groups.

**Fig. 4:**
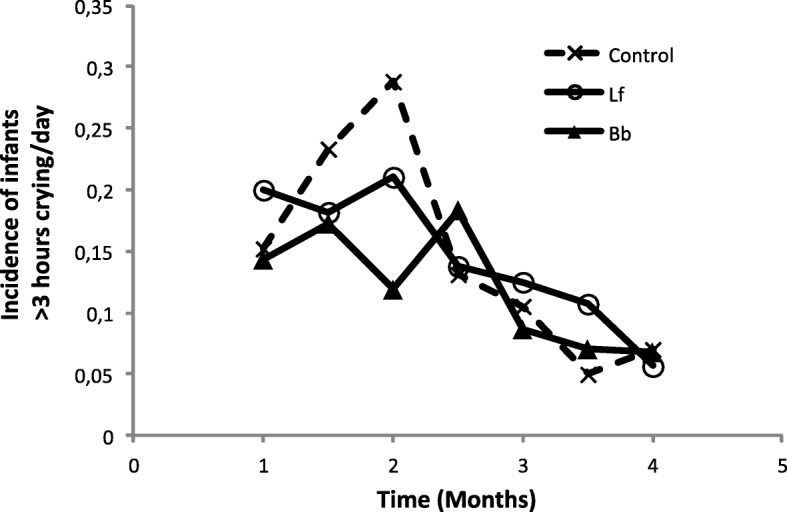
Incidence of infant crying > 3 h per day

Some variables related to the behaviour of infants were analysed. Data about the daily hours of crying were collected. In general, infants receiving *B. breve* CECT7263 had 1.7 times lower risk of long episodes of crying along the study than did infants in the Control group (OR = 0.569 CI 95% 0.568–0.571; *p* = 0.001). Because a symptom of infant colic is daily crying lasting more than 3 h, the frequency of infants crying more than 3 h was analysed (Fig. 4). At the beginning of the study, approximately 16% of infants cried more than 3 h/day. At 6 weeks, a maximum percentage of 29% was observed in the Control group, versus 21% in the Lf group (*p* = 0.335) and 12% in the Bb group (*p* = 0.022). The frequency of infants suffering from infant colic symptoms decreased to below 2% at 4 months of age, with no differences between groups after that age.

Hours of sleep during the night were similar in all the groups. The total hours of sleep per day were similar between the Bb group and the Control group (*p* = 0.927). The behaviour of the Lf group was different compared to the Control group (OR = 0.457; *p* = 0.028), but the effect was dependent on the age of the infants (p = 0.001). At the beginning of the study, infants in the Lf group had lower odds of sleeping more hours per day, but, at the end of the study, they had higher odds (OR = 2.828; *p* = 0.007). No differences were found in the level of activity of infants upon waking (*p* = 0.674 for Lf vs. Control group and *p* = 0.420 for Bb vs. Control group).

### Infant’s health

The most common infection during the first year of life of infants in this study was upper respiratory infections (83.4% of infants suffered at least one event). No differences were detected among the groups in incidence of respiratory infections affecting the upper or lower tract (Table 5). However, for infections affecting the upper respiratory tract, the adjusted results from the multivariate model showed an effect of smoking habits of the mothers during pregnancy in the Control group. Thus, the incidence of upper respiratory tract infections was significantly higher in cases of smoking women (IRR = 1.996 CI 95% 1.366–2.860; *p* = 0.000). In cases of infants from smoking mothers, the incidence of these respiratory infections in infants in the Lf and Bb groups was significantly lower than the incidence in the Control group (IRR = 0.503 CI 95% 0.274–0.899 and *p* = 0.023 for Lf group; IRR = 0.479; CI 95% 0.269–0.844 and *p* = 0.011 for Bb group). An effect of type of birth was also detected. In infants born by C-section the incidence of upper respiratory infections was lower in Lf group than in control group (IRR = 0.492; CI 95% 0.294–0.815; *p* = 0.006). In case of Bb group the effect did not reach to be statistically significant although a trend was observed (IRR = 0.630; CI 95% 0.372–1.062; *p* = 0.084).

**Table 5:** Incidence of infectious disease, febrile episodes and dermatitis during the intervention period

	Number of events (N)			Incidence Rate (Standard Error)			Lf vs Control group		Bb vs Control group	
	Control (61)	Lf group (65)	Bb group (63)	Control	Lf group	Bb group	IRR (95% CI)	*p-value*	IRR (95% CI)	*p-value*
Diarrhoea^a^	42	25	49	0.689 (0.106)	0.385 (0.077)	0.778 (0.113)	0.559 (0.326–0.938)	0.014	1.129 (0.745–1.777)	0.750
Upper tract	146	168	132	2.393 (0.212)	2.585 (0.206)	2.096 (0.188)	1.080 (0.829–1.309)	0.673	0.876 (0.663–1.076)	0.223
Lower tract	34	49	44	0.557 (0.101)	0.754 (0.108)	0.700 (0.107)	1.354 (0.814–2.054)	0.190	1.257 (0.756–1.953)	0.293
Conjunctivitis	11	4	8	0.180 (0.054)	0.062 (0.031)	0.127 (0.045)	0.344 (0.080–1.170)	0.071	0.705 (0.246–1.922)	0.473
Otitis	11	11	18	0.180 (0.054)	0.169 (0.051)	0.286 (0.067)	0.939 (0.369–2.387)	0.883	1.589 (0.709–3.712)	0.218
Urine Infection	2	2	1	0.033 (0.023)	0.031 (0.022)	0.016 (0.016)	0.940 (0.069–13.149)	0.958	0.485 (0.008–9.300)	0.540
Fever	32	28	27	0.525 (0.096)	0.431 (0.081)	0.429 (0.084)	0.821 (0.461–1.362)	0.193	0.817 (0.463–1.383)	0.172
Dermatitis	7	2	7	0.115 (0.043)	0.031 (0.022)	0.111 (0.042)	0.270 (0.028–1.430)	0.105	0.965 (0.290–3.235)	0.947

^a^Of the 42 diarrhea events in the control group, 19 occurred before 6 months and 23 in the period of 6 to 12 months. In the case of the Lf group, of the 25 diarrhea events 8 took place before 6 months and 17 in the period of 6 to 12 months

Regarding to gastrointestinal infections, 39% of the infants suffered at least one event of diarrhoea. Logistic regression mixed model analysis showed a general increase in the risk of diarrhoea throughout the study (*p* < 0.001) and 2.5 times higher odds of diarrhoea in infants attending kindergarten (*p* = 0.005). No differences were detected in the incidence of diarrhoea between the Bb group and the Control group. However, the consumption of *L. fermentum* CECT5716 significantly reduced the incidence of diarrhoea by 44% in comparison with the Control group (IRR 0.559; CI 95% 0.326–0.938; *p* = 0.014) (Table 5). Analysis including the data of all infants, while they had not completed the study, showed similar results (IRR = 0.587; CI 95% 0.351–0.961; *p* = 0.037). No differences were observed in the risks of having at least 1 event of diarrhoea (OR = 0.86; CI 95% 0.41–1.77; *p* = 0.732) but were observed in the risk of having more than 1 event of diarrhoea, which was 14 times lower in the Lf group than in the Control group (OR = 0.07; CI 95% 0–0.54; *p* = 0.002). The beneficial effect of *L. fermentum* CECT5716 on diarrhoea was also observed in the duration of the events. The mean duration of diarrhoea in the Control group was 7.10 ± 4.9 days (CI 95% 5.25–8.95), while in the Lf group, the mean duration of diarrhoea was 4.55 ± 3.6 days (CI 95% 2.95–6.14) (*p* = 0.044). No differences were observed in the duration of diarrhoea in the Bb group (6.24 ± 4.7 days; CI 95% 4.57–7.91; *p* = 0.482 in comparison with the Control group).

No significant differences were observed in the incidence of other infectious diseases (otitis, conjunctivitis and urinary tract infections), febrile episodes and dermatitis (Table 5).

#### Faecal microbiota

Some of the most representative bacterial genera were studied (Table 6). The abundance of *Lactobacillus* in faeces was higher for the first months and decreased with time (*p* = 0.000). Infants in the Lf group had significantly higher values of *Lactobacillus* in their faeces in comparison to the Control group (p = 0.000) and Bb group (*p* = 0.024). At 4 months of age, infants in the Bb group showed higher levels of *Lactobacillus* in their faeces (*p* = 0.000), but differences were not observed in later measurements. Regarding *Bifidobacterium*, a general increase over time was observed (*p* = 0.000). In the case of the Bb group, no differences were observed between bacterial load in faeces of infants in the Bb group and those of the Control group (*p* = 0.085). For the Lf group, a lower load of *Bifidobacterium* was observed at 4 months (*p* = 0.038), but no significant differences were observed at later times. A higher load of *Bifidobacterium* in faeces was related to a lower risk of diarrhoea (OR = 0.767 CI 95% 0.608–0.976; *p* = 0.027).

**Table 6:** Intestinal microbiota counts in faecal samples of infants (as log10 of cfu/g), at 4, 6, 9 and 12 months of age. Values are Mean + Standard Error of the Mean (SEM)

	*Control group*				*Group Lf*					*Group Bb*				
*Bacterial group*	4 month	6 months	9 months	12 month	4 months	6 months	9 months	12 months	p-value vs control	4 months	6 months	9 months	12 months	p-value vs control
*Lactobacillus spp.*	6.83 ± 0.1	7.12 ± 0.2	7.14 ± 0.1	6.58 ± 0.1	8.73 ± 0.1	8.39 ± 0.1	7.75 ± 0.1	7.21 ± 0.1	0.000	7.48 ± 0.1	7.29 ± 0.1	7.07 ± 0.1	6.45 ± 0.1	0.024
*Bifidobacterium spp.*	9.88 ± 0.1	9.91 ± 0.1	9.91 ± 0.1	9.93 ± 0.1	9.53 ± 0.1	9.77 ± 0.1	9.89 ± 0.1	9.78 ± 0.1	0.903	9.73 ± 0.1	9.91 ± 0.1	9.94 ± 0.1	9.67 ± 0.1	0.085
*Clostridium spp.*	5.38 ± 0.1	6.14 ± 0.1	6.64 ± 0.1	6.63 ± 0.1	5.64 ± 0.1	6.25 ± 0.1	6.72 ± 0.1	6.66 ± 0.1	0.632	5.71 ± 0.1	6.45 ± 0.1	6.59 ± 0.1	6.54 ± 0.1	0.026
*Bacteroides spp.*	6.59 ± 0.2	7.08 ± 0.1	7.68 ± 0.2	7.83 ± 0.2	6.44 ± 0.2	7.33 ± 0.2	8.03 ± 0.2	8.22 ± 0.2	0.044	6.04 ± 0.2	7.12 ± 0.2	7.39 ± 0.2	7.93 ± 0.2	0.777
*E.coli*	10.31 ± 0.2	9.97 ± 0.1	9.73 ± 0.1	9.51 ± 0.1	10.20 ± 0.1	10.07 ± 0.1	9.46 ± 0.1	9.16 ± 0.1	0.089	10.41 ± 0.1	9.96 ± 0.1	9.78 ± 0.1	9.47 ± 0.1	0.806

A general increase in *Clostridium* load in faeces was observed with time (p = 0.000). A significant difference was observed between the Bb group and the Control group, with infants in the Bb group having a higher level of *Clostridium* in their faeces (*p* = 0.026). *Clostridium* load in infants attending kindergarten was significantly lower (*p* = 0.009), but it did not change the effect of the treatment. It was observed that *Clostridium* load in faeces was significantly associated with a reduction of the risk for dermatitis for all groups (*p* = 0.010), meaning infants with higher levels of *Clostridium* had a lower risk of developing dermatitis. With respect to *Bacteroides,* increasing values of bacteria with time were observed in all groups (p = 0.000). Infants in the Lf group had higher values of *Bacteroides* than infants in the Control group (*p* = 0.044). A decrease in the load of *Escherichia coli* was also detected with time (p = 0.000). Values of *E. coli* in faeces were comparable among the groups (*p* = 0.806 for the Control group vs. Bb group; *p* = 0.089 for the Control group vs. Lf group).

## Discussion

The effects and safety of the two probiotic strains originally isolated from breast milk have been studied in the present trial in infants. Determination of growth of infants is the single most valuable component of the clinical evaluation of an infant formula [28, 29]. The analysis of the curves of growth for weight, length and head circumference by age showed similar values for the three groups. Moreover, the values of three groups were comparable for the standards of growth based on healthy infants published by the World Health Organization [30], indicating the nutritional sufficiency and safety of the experimental formula. Although, in general, the growth of infants was similar in the three groups, at 4 months a higher length was observed in infants in the Lf group. However, the difference was not detectable in the measurements carried out in the subsequent months. In previous studies, a certain effect of *L. fermentum* CECT5716 on the length of infants who received the probiotic strain up to 6 months of age was also observed [12]. Although, the difference was not sustained, and the length of the children at 3 years of age was similar to a control group [13]. The effect was not observed in a third study performed in infants who received the probiotic strain from 6 to 12 months of age [14]. The effects of probiotic strains on growth of infants have been observed for some strains [31, 32]. It has been proposed that the activity of the bacteria on mucosal physiology might influence the absorption of nutrients, as well as metabolic and endocrine functions [12, 33]. Even so, more studies should be performed in order to investigate the mechanisms involved. Interestingly, it was observed that the negative effect on length of C-section was counteracted by *L. fermentum* CECT5716 and *B. breve* CECT7263. C-section, which involves preventive antibiotic treatment for the woman, affects infant gut colonization [34]. It has been previously hypothesized that certain probiotics may prevent or attenuate the adverse effects of antibiotics on gut communities, thereby stabilizing gut integrity and improving the absorption of nutrients [33]. Therefore, the effects observed in our study might be related to the modulation of gut microbiota by the probiotic strains.

Continuing with the evaluation of the tolerance of the probiotic formula, the data regarding the daily intake of formula and the presentation of gastrointestinal symptoms, such as reflux, constipation or flatulence, demonstrated that both probiotic formulas were well tolerated.

Interestingly, during the evaluation of the behaviour of infants, it was detected that infants receiving the Bb formula had significantly lower risk of crying than did the control group. Infantile colic is a benign, self-limiting process in which a healthy infant has paroxysms of inconsolable crying. The standard diagnostic criteria is based in crying time more than three hours per day, more than three days per week, for longer than three weeks [35]. It affects approximately 10 to 40% of infants worldwide and peaks at around six weeks of age. Symptoms resolve usually by three to four months. In our study, a maximum percentage of infants crying more than 3 h/day was observed at 6 weeks (29%); however, this percentage was reduced to 12% in the group receiving the *Bifidobacterium* strain. Although the cause of infantile colic has not been totally elucidated, alterations in faecal microflora, intolerance to cow’s milk protein or lactose, gastrointestinal immaturity or inflammation, increased serotonin secretion, poor feeding technique, and even maternal smoking have been related [36]. In line with the role of the microbiota, some probiotic strains have been reported to reduce the crying time of infants suffering from infantile colic [37]. As the present study was performed with a generally healthy population, specific studies to evaluate the effect of *B. breve* CECT726 on infantile colic should be performed in order to corroborate the possible role of this strain in reducing the symptoms of this condition.

The most common infection during the first year of life in the infants of this study was upper respiratory infections. A beneficial effect was observed with both probiotic treatments in infants born from smoking mothers. This population, who are at a higher risk of suffering respiratory infections [38], showed lower rates of incidence in the groups consuming *L. fermentum* CECT5716 or *B. breve* CECT7163. A higher incidence of respiratory infections in infants born from smoking mothers has been related to the adverse effects of in utero smoke exposure on the infant’s immune system [39–41]. Because *Lactobacillus* and *Bifidobacterium* are not common habitants of upper respiratory mucosa, it seems that the effect of the probiotic strains on the immune system might contribute to the observed effect. In this sense, *L. fermentum* CECT5716 was previously described to reinforce the immune response by enhancing both the innate and specific immune responses [42, 43]. Recent findings have highlighted important roles of gut microbiota on lung immunity [44] and certain probiotic treatments have shown efficacy in the prevention of respiratory infections and/or reduction in the severity of the infections [45, 46]. The effect of *L. fermentum* CECT5716 on upper respiratory infections in infants was also previously observed in a study performed in infants with a follow-on formula containing the *Lactobacillus* strain. In that study, the consumption of the probiotic formula was related to a reduction in the incidence rate of upper respiratory infections by 26% in comparison with the incidence in infants receiving a standard formula [14]. A limitation of our study is that the effect has been observed in infants born from smoking mothers, which is a small proportion of the infants of the study.

*L. fermentum* CECT5716 has also been related to significant reductions in the incidence rates of gastrointestinal infections [12, 14]. In our study, a reduction of 44% in the incidence of diarrhoea has been observed. A limitation of the study is that the sample size was calculated in order to detect differences in the weight gain of infants as a safety marker. The sample size needed to detect differences in the incidence of gastrointestinal infections would be higher. However, the value of the decrease in incidence is very similar to the 46% observed in the Maldonado study [14]. Therefore, the results of the present study corroborate previous ones, showing that, in a repetitive and consistent way, the administration of this strain to infants being fed formula protects them against gastrointestinal infections. Different mechanisms, such as the competitive phenomena, production of antibacterial compounds and improvement of the immune response, have been attributed to the anti-infectious activity of probiotics and concretely to *L. fermentum* CECT5716 [47]. The effect of the strain seems to primarily affect repetitive infections, supporting a probable role of the immune system. The consumption of the probiotic strain also reduced the duration of diarrhoea by approximately 2.5 days with respect to the control group. There is extensive literature regarding the efficacy of probiotics on the treatment of diarrhoea. A systematic review, which included 56 trials, concluded that probiotics reduced the duration of diarrhoea by a mean of one day (24.76 h; 95% confidence interval 15.9 to 33.6 h; *n* = 4555); however, the size of the effect varied considerably among studies [48]. A larger difference in duration of diarrhoea has also been reported in another study performed in healthy infants receiving an infant formula containing a strain of *Bifidobacterium longum* [49]. More studies are needed to determine if probiotic use during childhood is a more efficient tool in reducing the severity of eventual diarrhoea than the treatment approach.

Regarding faecal microbiota, minor changes were associated with the probiotic treatments. As previously reported [14], the administration of *L. fermentum* CECT5716 was associated with an increase in the faecal load of lactobacilli. Moreover, a higher faecal load of *Bacteroidetes* was observed. On the other hand, intervention with *B. breve* CECT7263 did not affect the *Bifidobacterium* content in faeces. The concentration of *Bifidobacterium* in faeces is close to 10^10^ cfu/g, 3 magnitudes of order higher than *Lactobacillus*. Since the daily ingestion of *B. breve* was between 7 × 10^8^ and 1 × 10^9^ cfus, it would be difficult to observe significant changes in the total load of *Bifidobacterium* in faeces. However, it has been previously reported that while changes in *Bifidobacterium* spp. load could not be observed after oral administration of an infant formula supplemented with another *Bifidobacterium* strain, *B. animalis lactis* ssp. lactis Bb12, the strain could be detected in the faeces of a high percentage of infants who received the probiotic formula [50]. Therefore, while *B.breve* CECT7263 did not induce a significant increase in faecal *Bifidobacterium* population, the strain, included as one more species in the total microbiota, is able to affect the intestinal and immune functions as supported by the observed effects on infant colic and respiratory infections. The microbiota analysis in our study has been restricted to specific bacterial groups. In order to better understand how these probiotic strains might influence the microbiota of infants, a more complete microbiota analysis should be performed.

## Conclusion

Consumption of *L.fermentum* CECT5716 or *B.breve* CECT7263 during first year of life is well tolerated and safe. Additionally, beneficial effects of the strains consumption were observed. While *L. fermentum* CECT5716 stands out for its protective effects against gastrointestinal infections, *B. breve* CECT7263 stands out for its effects on the symptoms of infantile colic, which is probably related to its effect on intestinal function. Given that both strains are found naturally in breast milk and show beneficial activities that could complement each other, the combination of both strains in infant formulas could be used as a strategy to improve the health of formula-fed infants. Since this study was designed to demonstrate safety of the probiotic formulae, new clinical trials focused in each beneficial effect should be performed to confirm these results from the secondary outcomes.

## Data Availability

The datasets used and/or analysed during the current study are available from the corresponding author on reasonable request.

## Abbreviations

CI: Confidence Interval
IR: Incidence Rate
IRR: Incidence Rate Ratio
OR: Odd Ratio
